# Associations between activity, sedentary and sleep behaviours and psychosocial health in young children: a longitudinal compositional time-use study

**DOI:** 10.1186/s44167-022-00011-3

**Published:** 2023-01-03

**Authors:** Rachael W. Taylor, Jillian J. Haszard, Kim A. Meredith-Jones, Anita A. Azeem, Barbara C. Galland, Anne-Louise M. Heath, Barry J. Taylor, Dione Healey

**Affiliations:** 1 Department of Medicine, University of Otago, PO Box 56, Dunedin, New Zealand; 2 Biostatistics Centre, University of Otago, Dunedin, New Zealand; 3 Department of Psychological Medicine, University of Otago, Dunedin, New Zealand; 4 Department of Women’s and Children’s Health, University of Otago, Dunedin, New Zealand; 5 Department of Human Nutrition, University of Otago, Dunedin, New Zealand; 6 Department of Psychology, University of Otago, Dunedin, New Zealand

**Keywords:** Child, Physical activity, Sleep, Mental health, Wellbeing, Time-use epidemiology

## Abstract

**Background:**

Good quality sleep, regular physical activity, and limited time spent sedentary are all considered individually important in promoting good mental health in children. However, few studies have examined the influence of each behaviour simultaneously, using compositional analysis which accounts for the closed nature of the 24-h day. Our aim was to determine how compositional time use in early childhood is prospectively related to mental and psychosocial health at 5 years of age.

**Methods:**

A total of 392 children wore Actical accelerometers 24-h a day for one week at 2, 3.5 and 5 years of age to examine time in sleep, physical activity, and sedentary behaviour. Psychosocial and mental health were assessed at age 5 using both laboratory based (researcher-assessed) and questionnaire (parental-report) measures. Associations were estimated using regression models with isometric log-ratios of time-use components as predictors.

**Results:**

Cross-sectionally, 5-year old children who spent 10% (64 min) more time asleep than average had better inhibitory control (standardised mean difference [*d*]; 0.19; 95% confidence interal [CI]: 0.02, 0.36 for Statue test and *d* = 0.16; 95% CI: − 0.01, 0.33 for Heads–Toes–Knees–Shoulders task). A greater proportion of time spent active (10%, 31 min) was associated with poorer inhibitory control (*d* = − 0.07; 95% CI: − 0.13, − 0.02 for Statue test, *d* = − 0.06; 95% CI: − 0.11, − 0.01 for Heads–Toes–Knees–Shoulders task). By contrast, differences in time-use were not found to be significantly associated with any measure of self-regulation or mental health at 5 years of age, nor were any significant longitudinal relationships apparent.

**Conclusions:**

We did not find a significant association between 24-h time use in the preschool years and any measure of psychosocial or mental health at 5 years of age, although some relationships with inhibitory control were observed cross-sectionally.

*Trial registration*: ClinicalTrials.gov number NCT00892983, registered 5th May 2009.

## Introduction

Young children require positive environments to promote the development of good mental health, to develop resilience to cope with the stresses of life, and to grow into well-rounded, healthy adults [1]. While hesitancy exists around diagnosing very young children as having mental disorders, factors like improving social competence, emotional maturity, and physical wellbeing are known to play a crucial role in bolstering mental health [2]. It is important to consider wellbeing in children holistically, often expressed as psychosocial health which refers to psychological and social factors which contribute to overall mental health. It includes protective characteristics like high self-esteem, higher resilience, greater executive functioning and ability to regulate behaviour (including inhibitory control) as well as risk factors such as anxiety, depression, hyperactivity and attentional problems [3].

Within the physical health domain, ensuring that children receive sufficient good quality sleep, maintain regular activity, and limit sedentary time are all individually important [4–6]. However, because time spent in one behaviour will influence time spent in the remaining behaviours across a 24-h day, researchers are increasingly examining the health effects of these movement behaviours in concert rather than in isolation [7]. As a consequence, physical activity guidelines in many countries have expanded to also address sleep and sedentary time, renamed as 24-h guidelines [8–13].

To date, little research has determined how sleep, physical activity and sedentary time interact to influence mental health in preschool-aged children, a time when they are rapidly developing. Existing cross-sectional [14–16] and longitudinal [17–19] studies demonstrate little evidence that adherence to 24-h guidelines is associated with mental health at this age. However, a limitation of assessing health outcomes in this way is that it enforces an all or nothing approach by dichotomising continuous variables (i.e. meeting the guideline or not), resulting in a loss of information [20]. An alternative is to use compositional data analysis, which accounts for the fact that any health effects of changing one behaviour (e.g. getting more sleep) might be partly due to compensatory changes in the remaining behaviours (physical activity, sedentary time), rather than an isolated effect of sleep itself [21]. To date, only two small cross-sectional studies have examined compositional time use in relation to mental health in young children, showing some inconsistency in findings [22, 23]. Given the paucity of data, particularly longitudinal data, the aim of this study was to determine how compositional time use in early childhood is related to mental health outcomes at five years of age.

## Methods

This secondary analysis uses data from a randomised controlled trial of early-life obesity prevention (Prevention of Overweight in Infancy [POI] study) in Dunedin, New Zealand, consisting of a 2-year intervention (pre-natal to 2 years of age) [24] and 3-year follow-up (at 3.5 and 5 years of age) [25]. In brief, the interventions promoted the development of good sleep habits from birth (Sleep group), breastfeeding, healthy food and family activity (FAB group), or both (Combination group) in relation to Usual care. The current data have been analysed using the entire cohort with appropriate adjustment for randomisation group, as no significant differences were observed in physical activity, sedentary behaviour, or sleep following the intervention [26–28].

Detailed information regarding POI is available in registration documents (ClinicalTrials.gov number NCT00892983, registered 5th May 2009), study protocols [24, 25] and published findings [27, 28]. The intervention was approved by the Lower South Ethics Committee (LRS/12/08/063) and the follow-up study by the University of Otago Human Ethics Committee (12/274). Written informed consent was obtained from the parent/guardian of all child participants. We invited all mothers who had booked into the single maternity hospital (> 97% of all births) in Dunedin to participate when in the latter stages of their pregnancy. The final sample included 802 women (58% response rate) randomised to Usual care; Sleep; Food, Activity and Breastfeeding; or Combination groups. Anthropometric assessments (primary outcome) were performed by researchers blinded to group allocation. Demographic information obtained at baseline included maternal age, education, ethnicity, self-reported pre-pregnancy height and weight, and level of household deprivation. Information on infant gestational age, sex and birth weight was obtained from hospital records.

### Measures obtained at 2, 3.5 and 5 years of age

*Anthropometric* measurements were obtained by trained measurers following standard protocols [29]. Duplicate measures of weight (Tanita WB-100 MA/WB-110 MA) and height (Harpenden stadiometer, Holtain Ltd, UK) were obtained with children wearing light clothing. Body mass index (BMI) z-scores were calculated using the World Health Organization (WHO) growth standards [30], with overweight defined as a BMI z-score ≥ 85th but < 95th percentile, and obesity as BMI ≥ 95^th^ percentile.

*Sleep, physical activity, and sedentary behaviour* were assessed using Actical (Mini-Mitter, Bend, OR) accelerometers (initialized using 15 s epochs), worn by the children around the waist 24-h a day for one week. Data were scored using an automated count-scaled algorithm that estimates sleep onset (start of first 15 continuous minutes of sleep preceded by 5 min of awake) and offset (last of 15 continuous minutes of sleep followed by 5 min of awake) specific to each individual each day. Total sleep time was calculated as the difference between sleep onset and offset, excluding waking after sleep onset (WASO) as recommended [31]. Naps were determined in children at 1 and 2 years of age only defined as at least 30 min of continuous sleep, preceded by 5 min of being awake between 9am and 5 pm [32]. Awake time was divided into non-wear time (at least 20 min of consecutive zeros [33]), sedentary time (0–6 counts/15 s), light physical activity (LPA, 7–286 counts/15 s), and moderate-to-vigorous physical activity (MVPA, ≥ 287 counts/15 s) [34, 35]. We chose to use 20 min of consecutive zeros with no allowance for artifactual movement, similar to recommendations by Esliger et al. [33] and Janssen et al. [36]. Although allowing for some epochs above zero counts allows for touching or moving the accelerometer by accident and spurious spikes of accelerometer counts during non-wear without turning non-wear into sedentary time, allowing interruptions may also decrease classification accuracy [37] as well as making results vulnerable to variation in wear time if analysed with different epoch lengths [38]. As each 24-h ‘day’ was determined from the time the child woke up on day 1 to the time they woke up on day 2 (and so on), a day was considered valid if the participant had 20–28 h of data to allow for changing wake times. Participants had to have at least three valid days to be included in analyses (data from 161 children were excluded). Snoring at 3.5 and 5 years of age was adjusted for in analyses as it is one of the most common forms of sleep disturbance at this age [39], and can lead to problems with behavioural and emotional regulation [40]. Parents were asked ‘how often does your child snore loudly’ with 7 answer options ranging from ‘never’ to ‘every night’.

### Measures obtained at 5 years of age only

Psychosocial factors were measured using laboratory assessment and parental report. We determined levels of inhibitory control using the ‘Statue’ component of the NeuroPSYchological Assessment (NEPSY-2) [41] and the Head-Toes-Knees-Shoulders task [42]. NEPSY-2 is a test battery that is well-normed, reliable, and appropriate for use with 5-year-old children. The Head-Toes-Knees-Shoulders task [42] is a measure of behavioural regulation and inhibition, which determines the ability of a child to follow opposing instructions (e.g. touch the head when directed to touch the toes).

Parental ratings were obtained using the Parent Rating Scale of the Behavioral Assessment System for children (BASC-2), a well-validated and normed scale [43]. We used the ‘Hyperactivity’ (11 items, Cronbach’s α = 0.79), ‘Emotional Self-Control’ (6–8 items, Cronbach’s α = 0.79), ‘Executive Functioning’ (13 items, Cronbach’s α = 0.79) and ‘Attentional Problems’ (6 items, Cronbach’s α = 0.80) subscales as measures of self-regulation, and the ‘Anxiety’ (13 items, Cronbach’s α = 0.82), Depression (11 items, Cronbach’s α = 0.77), and ‘Resilience’ (12 items, Cronbach’s α = 0.82) subscales as indicators of the child’s mental health*.*

### Statistical analyses

Demographic characteristics were described for those with measures at 2, 3.5, or 5 years of age, and those who provided at least one measure (full analysis sample). Differences between the full analysis sample and the remaining POI participants were assessed using a independent samples t-test for continuous variables and a chi-squared test for categorical variables.

Time use components were normalised to sum to 24-h, with non-wear time first reallocated proportionally to wake-time components only (e.g. if the proportion of day-time wear is 80% sedentary and 20% PA, then 80% of the minutes of non-wear time are added to sedentary time and 20% of the minutes of non-wear time are added to PA). Non-wear time does not occur overnight so it is important that these minutes of non-wear time are not inadvertently assigned to sleep [44]. Compositional analyses were undertaken using a 3-component composition (sleep, sedentary, physical activity) because international guidelines for preschoolers focus on light-to-vigorous physical activity (LMVPA), and because most (80%) of our measurements at 5 years were obtain just prior to the child’s birthday. However, a 4-component composition (sleep, sedentary, light physical activity [LPA], and moderate-to-vigorous physical activity [MVPA]) was also undertaken for the cross-sectional analyses at 5 years, and longitudinal analyses using the 3.5 year data, based on the Level 2 Canadian guidelines for 3–4 year olds which recommend that at least 60 of the 180 min a day in LMVPA is spent in energetic play [8]. Compositional means for each component were calculated as geometric means normalised to 24 h [45]. To be able to include all co-dependent compositional variables in a regression model together, compositional data analysis (CoDA) methods were used. This involves using isometric log-ratios of the components, based on a sequential partition of one part to the remaining compositional parts [45], and including these coordinates in a linear regression model as the independent variables with the relevant outcome as the dependent variable. Models were adjusted for sex, household deprivation, randomisation group, and BMI z-score at 5 years of age, and snoring at 3.5 and 5 years of age as previously mentioned.

To report meaningful estimates of association between time-use components and mental or psychosocial health, the regression coefficient of the first isometric log-ratio coordinate (which contains the ratio of one component to all others) was back-transformed to represent the mean difference in the dependent variable for a 10% greater time spent in the component of interest relative to all others. Choosing to report associations in terms of a 10% difference is arbitrary but tends to represent a meaningful, yet still realistic difference in time-use (a 1% difference is not meaningful, while a 20% difference is less plausible). This is the most common way that associations with proportional differences are reported in studies of compositional time-use [46]. Separate regression models were generated to report estimates for each time-use component, with the isometric log-ratios calculated for different permutations of components.

Longitudinal associations between time use at 2 and 3.5 years and mental or psychosocial health at 5 years were assessed in the same way as the cross-sectional associations but without adjustment for 5-year time-use using linear regression models. All mental and psychosocial health variables were standardised so that estimates are presented in units of standard deviations. Standardised mean differences [*d*] and their 95% confidence intervals [CI] were calculated, estimating the mean difference for a 10% greater time in the component relative to all other components. Residuals of all regression models were plotted and visually assessed for homogeneity of variance and normality. All statistical analyses were carried out in Stata 17.0 (StataCorp, Texas).

## Results

Table 1 presents characteristics of the 392 children who provided data for this study. Half of the children were boys, and two-thirds of the mothers were university educated. Included mothers were 2.8 years older than those not included (33.0 vs 30.2; p < 0.001) and were more likely to have a university degree (67.0% vs 55.5%, p < 0.001). There were no meaningful differences in maternal BMI (mean age 25.2 vs 24.9 years, p = 0.403) or infant sex (50.3% vs 52.2% male, p = 0.583) between those who were included in the study and those who were not. Time spent in sleep, sedentary behaviour, and physical activity at each age, and mean (SD) values for each of the mental and psychosocial health outcomes examined are shown in Table 1.

**Table 1:** Demographic, time-use, mental wellbeing and adaptive skills, inhibitory control, and self-regulation variables (n = 392^a^)

	Full analysis sample	Those with time-use data at 2 years of age	Those with time-use data at 3.5 years of age	Those with time-use data at 5 years of age
n	392	197	266	348
Maternal age at child’s birth (y)				
Mean (SD)	33.0 (4.5)	33.2 (4.2)	33.1 (4.4)	32.9 (4.4)
Maternal parity, *n* (%)				
Primiparous	173 (44.1)	81 (41.1)	118 (44.4)	153 (44.0)
Multiparous	219 (55.9)	116 (58.9)	148 (55.6)	195 (56.0)
Maternal education, *n* (%)				
School only	37 (9.5)	17 (8.6)	24 (9.1)	32 (9.2)
Post-secondary	92 (23.5)	40 (20.3)	63 (23.8)	83 (23.9)
University degree or higher	262 (67.0)	140 (71.1)	178 (67.2)	233 (67.0)
Maternal pre-pregnancy BMI (kg/m^2^)				
Mean (SD)	25.2 (5.3)	25.0 (5.2)	25.3 (5.4)	25.2 (5.2)
Household deprivation, *n* (%)				
1–3 (Low)	149 (38.5)	81 (41.8)	108 (41.1)	131 (38.1)
4–7	165 (42.6)	80 (41.2)	111 (42.2)	146 (42.4)
8–10 (High)	73 (18.9)	33 (17.0)	44 (16.7)	67 (19.5)
Sex, *n* (%)				
Male	197 (50.3)	100 (50.8)	140 (52.6)	176 (50.6)
BMI z-score at 5 years				
Mean (SD)	0.46 (0.85)	0.41 (0.86)	0.43 (0.89)	0.46 (0.84)
Female	195 (49.7)	97 (49.2)	126 (47.4)	172 (49.4)
Snore, *n* (%)	–	–	16 (6.0)	18 (5.2)
Sleep (minutes)				
Mean (SD)	–	681 (50)	653 (38)	634 (36)
Sedentary behaviour (minutes)				
Mean (SD)	–	462 (49)	474 (51)	490 (54)
Light physical activity (minutes)				
Mean (SD)	–	–^b^	239 (41)	228 (40)
Moderate to vigorous physical activity (minutes)				
Mean (SD)	–	–^b^	73 (31)	88 (32)
Light to vigorous physical activity (minutes)				
Mean (SD)	–	297 (53)	313 (60)	316 (61)
*Outcome measures all at 5 years of age*				
Inhibitory control				
Heads–Toes–Knees–Shoulders [38]				
Mean (SD)	26.1 (18.4)	25.8 (17.6)	25.2 (18.3)	26.3 (18.4)
Statue (NEPSY-2) [37]				
Mean (SD)	17.3 (8.8)	17.2 (8.8)	16.8 (8.9)	17.5 (8.8)
Self-regulation (BASC-2) [39]				
Hyperactivity				
Mean (SD)	9.6 (3.9)	9.2 (4.0)	9.8 (4.0)	9.6 (4.0)
Emotional self-control				
Mean (SD)	18.5 (3.0)	18.8 (2.8)	18.4 (2.9)	18.6 (2.9)
Executive functioning				
Mean (SD)	27.2 (4.2)	27.6 (4.1)	27.1 (4.1)	27.3 (4.2)
Attentional problems				
Mean (SD)	6.3 (2.4)	6.2 (2.3)	6.4 (2.3)	6.3 (2.5)
Mental wellbeing (BASC-2) [39]				
Anxiety				
Mean (SD)	9.1 (4.7)	8.5 (4.5)	9.0 (4.6)	9.0 (4.7)
Depression				
Mean (SD)	7.4 (3.5)	7.0 (3.3)	7.5 (3.5)	7.3 (3.5)
Resilience				
Mean (SD)	25.7 (4.7)	26.2 (4.8)	25.6 (4.6)	25.7 (4.7)

*BMI*  body mass index, *NEPSY-2*  NeuroPSYchological Assessment, *BASC-2*  Behavioral Assessment System for children

^a^n = 392 is the number of participants who had time-use data at either 2, 3.5 or 5 years of age and complete mental health/self-regulation data at 5 years of age. One participant missing maternal education; two missing maternal BMI, five missing household deprivation

^b^Not calculated as guidelines at this age refer to light-to-vigorous activity (LMVPA) only

Table 2 presents the cross-sectional associations illustrating how 5-year-old children spend their time using a model with the three-component composition (sleep, sedentary, light-to-vigorous physical activity) in relation to the outcomes measured. These data illustrate that children who spent 10% more sleep time than average (relative to all other time-use components), corresponding to an additional 64 min a night, had better inhibitory control as measured by the Statue test (*d* = 0.19; 95% CI: 0.02, 0.36) and the Heads–Toes–Knees–Shoulders task (*d* = 0.16; 95% CI: − 0.01, 0.33). By contrast, a greater proportion of time spent physically active was associated with lower inhibitory control, whether measured by the Statue test (*d* = 0.07; 95% CI: − 0.13, − 0.02) or the Heads–Toes–Knees–Shoulders task (*d* = − 0.06; 95% CI: − 0.11, − 0.01). Differences in time-use were not meaningfully associated with any measure of self-regulation or mental health at 5 years of age.

**Table 2:** Cross-sectional associations between 3-part time-use composition and inhibitory control, self-regulation, and mental wellbeing at 5 years of age (n = 344)

	Standardised mean difference (95% CI) in outcome for 10% more sleep time than average	Standardised mean difference (95% CI) in outcome for 10% more sedentary time than average	Standardised mean difference (95% CI) in outcome for 10% more physical activity time than average
	Sleep	Sedentary	Physical activity
Compositional mean (minutes)	637	490	313
10% of compositional mean (minutes)	64	49	31
Inhibitory control			
Heads–Toes–Knees–Shoulders [38]	0.16 (− 0.01, 0.33)	− 0.05 (− 0.15, 0.06)	− 0.07 (− 0.13, − 0.02)
Statue (NEPSY-2) [37]	0.19 (0.02, 0.36)	− 0.08 (− 0.19, 0.02)	− 0.06 (− 0.11, − 0.01)
Self-regulation (BASC-2) [39]			
Hyperactivity	0.07 (− 0.10, 0.23)	− 0.08 (− 0.18, 0.03)	0.02 (− 0.04, 0.07)
Emotional self-control	− 0.01 (− 0.18, 0.16)	0.01 (− 0.10, 0.12)	0.00 (− 0.06, 0.05)
Executive functioning	− 0.09 (− 0.26, 0.07)	0.06 (− 0.05, 0.17)	0.02 (− 0.04, 0.07)
Attentional problems	0.14 (− 0.03, 0.30)	− 0.09 (− 0.20, 0.01)	− 0.02 (− 0.07, 0.04)
Mental wellbeing (BASC-2) [39]			
Anxiety	0.05 (− 0.12, 0.22)	0.01 (− 0.09, 0.12)	− 0.05 (− 0.10, 0.01)
Depression	0.06 (− 0.11, 0.23)	− 0.02 (− 0.13, 0.09)	− 0.03 (− 0.08, 0.03)
Resilience	− 0.07 (− 0.24, 0.10)	0.01 (− 0.10, 0.11)	0.04 (− 0.01, 0.10)

All analyses adjusted for sex, deprivation, snoring, BMI z-score, and randomised group and analysed using compositional analysis that takes into account all time-use variables. Mean differences are for a 10% greater time spent in the component relative to all other components

*NEPSY-2* NeuroPSYchological Assessment, *BASC-2* Behavioral Assessment System for children

Similar analyses were undertaken at 5 years of age using a model with the 4-part composition (physical activity divided into light PA and moderate-to-vigorous PA, Table 3). These analyses were broadly similar to those observed with the three-part composition; we observed greater inhibitory control in those who had more sleep, and the lower scores for inhibitory control seen with greater time spent physically active were entirely a result of more light PA rather than MVPA. However, some differences in mental wellbeing were observed with spending 10% more time (8.4 min) each day in MVPA; children had lower anxiety (*d* = − 0.05; 95% CI: − 0.09, − 0.02) and higher resilience (*d* = 0.05; 95% CI: 0.01, 0.08) scores, albeit by a small amount. A lower score for attentional problems (*d* = − 0.12; 95% CI: − 0.23, 0.00) was also observed with 10% more time spent sedentary (49 min more).

**Table 3:** Cross-sectional and longitudinal associations between time-use and inhibitory control, self-regulation, and mental wellbeing at 5 years of age

	Standardised mean difference (95% CI) in outcome at 5 years for 10% more sleep time than average	Standardised mean difference (95% CI) in outcome at 5 years for 10% more sedentary time than average	Standardised mean difference (95% CI) in outcome at 5 years for 10% more light physical activity time than average	Standardised mean difference (95% CI) in outcome at 5 years for 10% more moderate to vigorous physical activity time than average
Cross-sectional associations: time-use at 5 years (n = 344)				
	Sleep	Sedentary	LPA	MVPA
10% of compositional mean (minutes)	64	49	23	8.4
Inhibitory control				
Heads–Toes–Knees–Shoulders [38]	0.16 (− 0.02, 0.34)	− 0.02 (− 0.14, 0.10)	− 0.11 (− 0.17, − 0.04)	0.02 (− 0.02, 0.05)
Statue (NEPSY-2) [37]	0.18 (0.00, 0.37)	− 0.05 (− 0.17, 0.07)	− 0.12 (− 0.18, − 0.06)	0.03 (− 0.00, 0.07)
Self-regulation (BASC-2) [39]				
Hyperactivity	0.08 (− 0.10, 0.26)	− 0.08 (− 0.20, 0.03)	0.01 (− 0.05, 0.07)	0.00 (− 0.03, 0.04)
Emotional self-control	− 0.02 (− 0.21, 0.16)	0.03 (− 0.09, 0.15)	− 0.04 (− 0.11, 0.02)	0.03 (− 0.01, 0.06)
Executive functioning	− 0.11 (− 0.30, 0.07)	0.08 (− 0.04, 0.20)	− 0.01 (− 0.08, 0.05)	0.02 (− 0.01, 0.06)
Attentional problems	0.16 (− 0.02, 0.33)	− 0.12 (− 0.23, − 0.00)	0.02 (− 0.05, 0.08)	− 0.02 (− 0.06, 0.01)
Mental wellbeing (BASC-2) [39]				
Anxiety	0.08 (− 0.11, 0.26)	− 0.02 (− 0.14, 0.10)	0.03 (− 0.04, 0.09)	− 0.05 (− 0.09, − 0.02)
Depression	0.08 (− 0.10, 0.27)	− 0.05 (− 0.17, 0.07)	0.02 (− 0.04, 0.09)	− 0.04 (− 0.07, 0.00)
Resilience	− 0.09 (− 0.28, 0.09)	0.04 (− 0.08, 0.16)	− 0.02 (− 0.09, 0.04)	0.05 (0.01, 0.08)

Longitudinal associations: time-use at 3.5 years (n = 263)				
	Sleep	Sedentary	LPA	MVPA
10% of compositional mean (minutes)	66	48	24	6.7
Inhibitory control				
Heads–Toes–Knees–Shoulders [38]	0.09 (− 0.12, 0.30)	− 0.01 (− 0.15, 0.12)	− 0.06 (− 0.13, 0.01)	0.02 (− 0.01, 0.04)
Statue (NEPSY-2) [37]	− 0.11 (− 0.32, 0.09)	0.09 (− 0.04, 0.22)	− 0.03 (− 0.10, 0.04)	0.02 (0.00, 0.05)
Self-regulation (BASC-2) [39]				
Hyperactivity	0.01 (− 0.18, 0.21)	− 0.05 (− 0.17, 0.08)	− 0.01 (− 0.07, 0.06)	0.03 (0.00, 0.06)
Emotional self-control	0.02 (− 0.19, 0.23)	− 0.01 (− 0.14, 0.13)	0.02 (− 0.05, 0.09)	− 0.02 (− 0.05, 0.01)
Executive functioning	− 0.04 (− 0.24, 0.16)	0.03 (− 0.10, 0.16)	0.04 (− 0.03, 0.11)	− 0.04 (− 0.06, − 0.01)
Attentional problems	0.06 (− 0.13, 0.26)	− 0.06 (− 0.19, 0.06)	0.03 (− 0.04, 0.09)	− 0.01 (− 0.04, 0.01)
Mental wellbeing (BASC-2) [39]				
Anxiety	− 0.04 (− 0.25, 0.17)	0.03 (− 0.10, 0.17)	− 0.02 (− 0.09, 0.05)	0.01 (− 0.01, 0.04)
Depression	− 0.05 (− 0.26, 0.16)	0.08 (− 0.05, 0.21)	− 0.06 (− 0.13, 0.01)	0.03 (− 0.00, 0.05)
Resilience	0.00 (− 0.21, 0.20)	− 0.03 (− 0.16, 0.10)	0.05 (− 0.02, 0.12)	− 0.02 (− 0.05, 0.01)

All analyses adjusted for sex, deprivation, snoring, BMI z-score, and randomised group and analysed using compositional analysis that takes into account all time-use variables. Mean differences are for a 10% greater time spent in the component relative to all other components

*NEPSY-2*  NeuroPSYchological Assessment, *BASC-2*  Behavioral Assessment System for children

Table 4 presents the same analyses using the longitudinal data, determining how time use at 2 and 3.5 years of age was related to mental health and wellbeing outcomes at 5 years of age. As Table 4 illustrates, different proportions of time spent in sleep, physical activity, or being sedentary at 2 or 3.5 years was not significantly associated with any measure examined at 5 years of age. Longitudinal analyses were also undertaken using the 4-component model (Table 3). Small differences were observed for greater time spent in MVPA. Spending more time in MVPA at 3.5 years of age was related to higher scores for hyperactivity (0.03; 0.00, 0.06) and lower scores for executive functioning (− 0.04; − 0.06, − 0.01) at 5 years.

**Table 4:** Longitudinal associations between three-component time-use at 2 and 3.5 years and inhibitory control, self-regulation, and mental wellbeing at 5 years of age

Outcomes	Standardised mean difference (95% CI) in outcome at 5 years for 10% more sleep time than average	Standardised mean difference (95% CI) in outcome at 5 years for 10% more sedentary time than average	Standardised mean difference (95% CI) in outcome at 5 years for 10% more physical activity time than average
Time-use at 2 years (n = 194)	Sleep	Sedentary	Physical activity
Compositional mean (minutes)	684	463	293
10% of compositional mean (minutes)	68	46	29
Inhibitory control			
Heads–Toes–Knees–Shoulders [38]	0.06 (− 0.10, 0.23)	− 0.06 (− 0.18, 0.05)	0.01 (− 0.06, 0.08)
Statue (NEPSY-2) [37]	0.02 (− 0.15, 0.20)	− 0.04 (− 0.16, 0.08)	0.02 (− 0.05, 0.09)
Self-regulation (BASC-2) [39]			
Hyperactivity	0.12 (− 0.05, 0.29)	− 0.08 (− 0.19, 0.04)	− 0.01 (− 0.08, 0.05)
Emotional self-control	− 0.08 (− 0.25, 0.08)	0.06 (− 0.06, 0.17)	0.01 (− 0.06, 0.07)
Executive functioning	− 0.10 (− 0.26, 0.07)	0.05 (− 0.06, 0.17)	0.02 (− 0.05, 0.08)
Attentional problems	− 0.03 (− 0.19, 0.12)	0.04 (− 0.06, 0.15)	− 0.02 (− 0.08, 0.05)
Mental wellbeing (BASC-2) [39]			
Anxiety	0.11 (− 0.06, 0.27)	− 0.04 (− 0.15, 0.08)	− 0.04 (− 0.10, 0.03)
Depression	0.10 (− 0.07, 0.26)	− 0.04 (− 0.15, 0.08)	− 0.03 (− 0.10, 0.03)
Resilience	− 0.11 (− 0.28, 0.07)	0.05 (− 0.07, 0.17)	0.03 (− 0.04, 0.10)

Time-use at 3.5 years (n = 263)	Sleep	Sedentary	LPA
Compositional mean (minutes)	656	475	309
10% of compositional mean (minutes)	66	48	31
Inhibitory control			
Heads–Toes–Knees–Shoulders [38]	0.09 (− 0.10, 0.28)	− 0.04 (− 0.16, 0.08)	− 0.03 (− 0.09, 0.03)
Statue (NEPSY-2) [37]	− 0.09 (− 0.28, 0.10)	0.06 (− 0.06, 0.18)	0.01 (− 0.05, 0.07)
Self-regulation (BASC-2) [39]			
Hyperactivity	0.03 (− 0.15, 0.21)	− 0.06 (− 0.18, 0.04)	0.04 (− 0.02, 0.09)
Emotional self-control	0.01 (− 0.18, 0.20)	0.01 (− 0.10, 0.13)	− 0.02 (− 0.08, 0.04)
Executive functioning	− 0.05 (− 0.23, 0.13)	0.06 (− 0.05, 0.18)	− 0.02 (− 0.08, 0.04)
Attentional problems	0.04 (− 0.13, 0.22)	− 0.04 (− 0.15, 0.07)	0.00 (− 0.05, 0.06)
Mental wellbeing (BASC-2) [39]			
Anxiety	− 0.03 (− 0.22, 0.16)	0.02 (− 0.10, 0.14)	0.01 (− 0.05, 0.07)
Depression	− 0.03 (− 0.23, 0.16)	0.04 (− 0.08, 0.16)	− 0.01 (− 0.07, 0.05)
Resilience	− 0.01 (− 0.20, 0.17)	0.00 (− 0.12, 0.12)	0.01 (− 0.05, 0.07)

All analyses adjusted for sex, deprivation, snoring (for the 3.5 year measures, not available for the 2 year measures), BMI z-score at 5 years, and randomised group and analysed using compositional analysis that takes into account all time-use variables. Mean differences are for a 10% greater time spent in the component relative to all other components

*NEPSY-2*  NeuroPSYchological Assessment, *BASC-2*  Behavioral Assessment System for children

## Discussion

Our findings show that young children who spend more time asleep have higher levels of inhibitory control, whereas children with greater levels of physical activity have lower inhibitory control, as a result of more time spent in light rather than more intense levels of activity. However, these relationships were not apparent longitudinally, with 24-h time use at 2 or 3.5 years not found to be significantly related to levels of inhibitory control, nor indeed any other measure of psychosocial or mental health at 5 years of age. Findings were broadly comparable, whether determined for the 4-part composition (examines physical activity separated into LPA and MVPA), or the 3-part composition (combines both components), with the following exceptions. Spending more time in MVPA was associated with lower anxiety and higher resilience scores cross-sectionally but also with lower executive functioning and higher hyperactivity longitudinally. Such relationships were not apparent when all categories of physical activity intensity (light, moderate, and vigorous) were combined.

Our finding that sleep was cross-sectionally related to measures of inhibitory control at 5 years of age, but that sleep at earlier ages did not predict later levels of control, fits much of the existing literature. A cross-sectional study in 3–5 year old children reported positive correlations between sleep duration and levels of inhibitory control as measured by a computerized go/no-go test [47]. Such findings have been confirmed through a meta-analysis of 86 predominantly cross-sectional studies in older (5–12 years) children demonstrating that reducing sleep duration compromised overall executive functioning, including inhibitory control [48]. By contrast, while some longitudinal studies have reported that night-time sleep at 12–18 months was proportionately related to strong impulse control at ages 2 and 4 [49, 50], others have not observed any longitudinal association with measurements taken at 4–7 and 9–16 years [51]. However, experimental research has demonstrated extending sleep by just half an hour a night enhances attention and inhibition in children, at least in the short term [52]. Overall, it would seem that sufficient levels of sleep, independent of sedentary time and physical activity, are advantageous for the development of appropriate inhibitory control in young children. However, it should be acknowledged that the differences observed were small, with wide CIs.

Our results also indicate an association between greater physical activity and lower inhibitory control in 5 year olds as a result of more time spent in light activity. These results, although complex, potentially occur because light activity may not be challenging enough to stimulate inhibition skills. Current literature suggests there needs to be an increment in task difficulty in order to improve cognitive functioning [53], and it seems likely that light activity does not produce the same benefit. Previous research in adolescents supports this view showing that light activity predicted lower performance on cognitive tasks whereas MVPA was associated with greater executive functioning [54]. Our study reports the same trends in children at 5 years of age. In line with other findings [55], we also found that higher time spent in MVPA was associated with lower anxiety and greater resilience. Finally, our finding that higher MVPA was associated with higher scores for hyperactivity is similar to other longitudinal studies [56, 57]. As these researchers highlight, there is potential for residual confounding and the possibility that some hyperactive symptoms appear as MVPA. The latter could also help explain the association between MVPA and lower executive functioning, given that this subscale contains five of the items also found within the hyperactivity subscale. Alternatively, variation in findings is possible due to the use of different cutpoints in the literature for delineating intensity of activity, which may have influenced the relationships observed.

Our study has several strengths including the relatively large sample size and longitudinal study design. We also measured 24-h time use with acceleromety rather than a mixture of measures, which has frequently been used in the compositional analysis literature to date. The use of 24-h accelerometry limits the amount of missing (or overlapping) data, problems that arise particularly when multiple methods are combined to assess 24-h time use. Our study had repeat measures of time use, allowing us to examine predictive relationships between time use and psychosocial health over an important stage of development. We also had both questionnaire and objective outcome measures; parental-report providing an overview of ‘usual’ psychosocial health, and laboratory-based assessments providing an objective and independent assessment, albeit only for a snapshot in time.

Our study also had some limitations. Only 392 of the original 802 (49%) children had sufficient data to be included in these analyses. However, this remains a substantial sample size and while there were some slight demographic differences between those who were included and those who were lost to follow-up, these were included as covariates in the models. As some participants did not complete accelerometry at every age, the samples analysed at each age also differed slightly—although demographically they were similar. While there was high variance in the outcome variables, the analysis sample is unlikely to be representative of the New Zealand population and associations could differ by demographic or at-risk groups.

## Conclusions

We found no evidence that variation in 24-h time use in preschoolers is associated longitudinally or cross-sectionally with any measure of self-regulation or psychosocial health at 5 years of age. Children who spend more time in sleep and those who spend less time being physically active, did show higher levels of inhibitory control, but differences were small. Our findings support a number of studies that have investigated this question in a different way, by examining adherence to 24-h guidelines in relation to mental health, which have also demonstrated little evidence of any relationship in pre-schoolers [14–19]. While absence of evidence is not the same as evidence of absence, the data to date demonstrate little support for the presence of meaningful relationships. Perhaps the discrepancy between literature examining the association with a single one of these behaviours in relation to mental health (which often supports a link) may be at least in part explained by the fact that examining any one behaviour (e.g. sleep) ignores the dependence on the remaining behaviours (physical activity, sedentary time); only compositional analyses can account for this correctly [21].

## Data Availability

The datasets used and/or analysed during the current study are available from the corresponding author on reasonable request.

## Abbreviations

MVPA: Moderate-to-vigorous physical activity
POI: Prevention of Overweight in Infancy study
BMI: Body mass index
WHO: World Health Organisation
WASO: Wake after sleep onset
NEPSY-2: NeuroPSYchological Assessment
BASC-2: Behavioral Assessment System for Children
